# Beneficial Effects of Plant-Based Diets on Skin Health and Inflammatory Skin Diseases

**DOI:** 10.3390/nu15132842

**Published:** 2023-06-22

**Authors:** Ximena Flores-Balderas, Mario Peña-Peña, Karla M. Rada, Yamnia Q. Alvarez-Alvarez, Carlos A. Guzmán-Martín, José L. Sánchez-Gloria, Fengyang Huang, Dayanara Ruiz-Ojeda, Sofía Morán-Ramos, Rashidi Springall, Fausto Sánchez-Muñoz

**Affiliations:** 1 Departamento de Inmunología, Instituto Nacional de Cardiología Ignacio Chávez, Mexico City 14080, Mexico; ximena_9814@hotmail.com (X.F.-B.); marionutricion2017@gmail.com (M.P.-P.); michelrp36@gmail.com (K.M.R.); gmcarlos93@gmail.com (C.A.G.-M.); jose_sanchez@rush.edu (J.L.S.-G.); daayziur@gmail.com (D.R.-O.); raspringall@yahoo.com (R.S.); 2 Sección de Estudios de Posgrado, Escuela Superior de Medicina, Instituto Politécnico Nacional, Mexico City 11340, Mexico; yamniaalvarezalvarez@gmail.com; 3 Departamento de Fisiopatología Cardiorrenal, Instituto Nacional de Cardiología Ignacio Chávez, Mexico City 14080, Mexico; 4 Laboratorio de Investigación en Obesidad y Asma, Hospital Infantil de México Federico Gómez, Mexico City 06720, Mexico; huangfengyang@gmail.com; 5 Unidad de Genόmica de Poblaciones Aplicada a la Salud, Facultad de Química, UNAM/Instituto Nacional de Medicina Genόmica (INMEGEN), Mexico City 14609, Mexico; sofi_moran@yahoo.com.mx; 6 Departamento de Alimentos y Biotecnología, Facultad de Química, Universidad Nacional Autónoma de México (UNAM), Mexico City 04510, Mexico

**Keywords:** diet, skin health, inflammatory skin diseases, plant-based diet, plant-based functional foods, psoriasis, atopic dermatitis, acne, microbiota

## Abstract

The human skin is a crucial organ that protects the organism from the outer environment. Skin integrity and health depend on both extrinsic and intrinsic factors. Intrinsic factors such as aging and genetic background contribute to weakened skin and disease susceptibility. Meanwhile, extrinsic factors including UV radiation, pollution, smoking, humidity, and poor diet also affect skin health and disease. On the other hand, healthy dietary patterns such as plant-based diets have gained popularity as a complementary therapy for skin health. A plant-based diet is defined as all diets based on plant foods, including an abundance of vegetables, fruits, beans, lentils, legumes, nuts, seeds, fungi, and whole grains, with limited or no animal products or processed foods. However, some authors also exclude or limit processed foods in the definition. Recent research has shown that these diets have beneficial effects on inflammatory skin diseases. This review explored the beneficial effects of plant-based diets on inflammatory skin diseases and plant-based functional foods on healthy skin. In conclusion, plant-based diets and plant-based functional foods may have beneficial effects on skin health through the gut microbiome.

## 1. Introduction

The skin is a vital organ that is exposed to the outer environment; thus, it has numerous functions such as body temperature regulation, protection against infections, and preservation of internal organs by acting as a protective barrier [1]. Skin damage can be caused by intrinsic and extrinsic factors. Intrinsic factors through genetic factors generate physiological skin aging, dry skin, wrinkles, dermal atrophy, angiogenesis, and increased skin permeability and affect negatively immune function and sweat production [2,3]. On the other hand, extrinsic factors include UV radiation, pollution, smoking, humidity, and poor diet [4]. Furthermore, there are various chronic conditions that can impact skin health, with inflammatory skin diseases being the most prevalent among them [5,6]. These skin diseases have a pharmacological well-established treatment such as immunosuppressant drugs, although most patients do not totally recover or may present adverse effects. Thus, patients are frequently seeking natural treatments both topical and non-topical. Dietary changes such as plant-based dietary patterns have gained popularity as complementary for therapy for skin diseases [7].

A plant-based diet is a dietary pattern composed of a large variety of vegetables, fruits, beans, lentils, legumes, nuts, seeds, fungi, and whole grains and limited or no intake of animal products, processed foods, or sweets [8]. American Dietetic Association poses that “appropriately planned vegetarian diets, including total vegetarian or vegan diets, are healthful, nutritionally adequate, and may provide health benefits in the prevention and treatment of certain diseases. Well-planned vegetarian diets are appropriate for individuals during all stages of the life cycle, including pregnancy, lactation, infancy, childhood, and adolescence, and for athletes” [9]. Plant-based diets have beneficial effects modulating inflammatory and oxidative processes, which are the main mechanisms in inflammatory skin diseases [10]. Additionally, they encompass a variety of fruits and vegetables classified as plant-based functional foods, which harbor additional components that can potentially contribute to overall health benefits [11,12,13].

**Figure 1:**
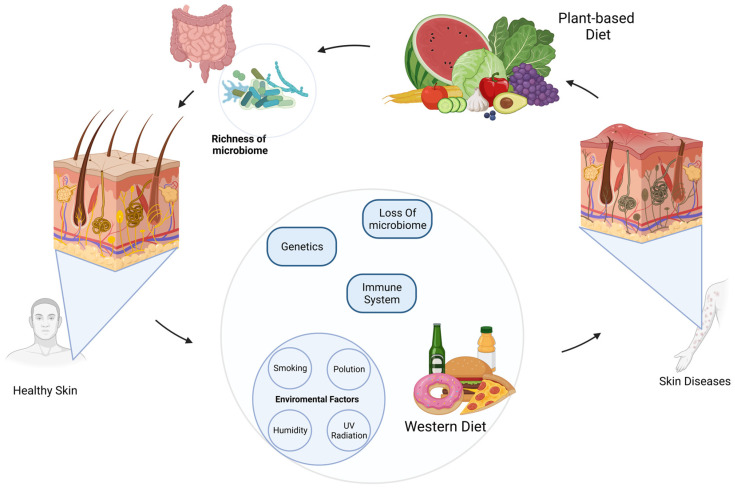
Association between plant-based diet and health/diseases of the skin. Skin health is influenced by the immune system, genetic predisposition, and environmental factors such as UV radiation, smoking, pollution, humidity, and diet. A Western diet has been shown to negatively impact skin health by disrupting the microbiome and leading to skin diseases. Conversely, a plant-based diet has been associated with healthier skin due to its ability to enrich and modulate the microbiome through various plant-based dietary patterns.

The focus of this review is to examine the impact of a plant-based diet and plant-based functional foods on promoting healthy skin and its positive effects on skin diseases (Figure 1). Moreover, this review examines the gastrointestinal link between the gut microbiome and plant-based diets in relation to skin health. This connection, referred to as the gut–skin axis, has been associated with various inflammatory and autoimmune skin conditions, including acne and psoriasis.

## 2. Association between Plant-Based Diet and Psoriasis

Psoriasis is defined as a chronic immune-mediated skin disease with characteristic scaly, erythematous plaques with well-defined-margin lesions [14]. In 2021, more than 7.5 million United States adults with psoriasis were registered, with a prevalence of 3% [15]. For many years, psoriasis was considered only a skin disease; however, it is now known that it is also a systemic pathology, with a persistent inflammatory state, that affects multiple organs and therefore results in a significant decrease in the quality of life [16]. Generally, the pathophysiological mechanisms underlying psoriasis involve a dysregulation of the immune response and epidermic cell differentiation [17]. Moreover, in psoriatic patients have been found elevated levels of inflammatory mediators such as interleukins and tumor necrosis factor-alpha TNF-a, which are the most characteristic circulating inflammatory markers in psoriasis [18]. A variety of environmental factors are involved in the pathogenesis of psoriasis and its comorbidities such as stress, smoking, and diet. Particularly, psoriasis is closely related to diet-related pathologies: obesity, metabolic syndrome, and cardiovascular disease [19].

Nowadays, patients with psoriasis are in search of complementary or alternative therapeutics such as natural methods to improve symptoms and for general health, so that they can improve their quality of life [20]. Currently, it is well described that certain diets have several benefits in psoriasis. In this regard, a cohort study investigated dietary behaviors in patients with psoriasis in which a 61-question survey was applied to 1206 patients in the US. The results reported that 86% of all respondents made changes to their diet, noting that the best positive results on skin improvement were observed with the vegan, Pagano, and Paleolithic diets compared to the effects obtained with the gluten-free, Mediterranean, vegetarian, and carbohydrate-high protein diets [21]. Dairy and sugar consumption was reported as one of the most common triggers for psoriasis, while meat and eggs were included as one of the minor common triggers [21]. Moreover, they observed that only adding vegetables to the habitual diet also had a favorable skin response in 42.5% of patients, probiotics in 40.6%, organic foods in 38.4%, and fruits in 34.6%. On the other hand, it was also observed that avoiding certain foods such as junk foods (50%), white flour products (49.9%), dairy (47.7%), pork (35.6%), and red meat (30.4%) had similar results. In addition, more than half of patients (69%) who followed any of these types of diets reported significant weight loss, showing a relationship between plant-based diets and a reduction in the BMI (Body Mass Index) [21]. Thus, extensive evidence has shown that specific diets, such as the vegan diet, decrease some symptoms of psoriasis, although the study does not mention what the specific changes were in patients with psoriasis. Beneficial effects on the skin are generally reported; however, more studies are necessary to understand the role of plant-based diets in disease management.

Overweight and obesity are indisputable risk factors for the development of psoriasis and have been documented as factors for severe stages of the disease [22]. The role of dietary interventions around psoriasis was investigated in a randomized controlled trial (N = 303) which reported the effects of a low-energy diet associated with physical exercise on the severity of psoriasis in patients with an elevated BMI that recently started treatment and whose response was not favorable with conventional systemic therapy with etanercept, infliximab, adalimumab, ciclosporin, and PUVA (8-MOP) psoralen combined with ultraviolet A therapy (8-methoxypsoralen) [23]. The results showed a decrease of 48% in the PASI (Psoriasis Area and Severity Index) score. This positive effect was higher in men, patients over 50 years old with a BMI between 25 and 29.9 kg/m^2^, and patients with PASI scores greater than or equal to 10. In this study, the intervention follow-up period of 20 weeks showed that 29.8% of subjects reached a significant weight loss of close to 5%. During the study, there were no major side effects surrounding the low-energy diet period, such as vitamin deficiencies; however, a minority of patients reported a sensation of hunger and constipation [23]. It is well known that an increase in the adipose tissue in obesity tends to produce excessive amounts of proinflammatory cytokines which participate in the pathophysiology of psoriasis, including IL-8, IL-6, and TNF [22]. Thus, if plant-based diets such as the vegan diet significantly decrease the BMI, and weight loss has a strong association with clinical improvement in psoriasis patients, it would be reasonable to suggest that these diets could be fundamental pillars for the management of psoriasis. However, the affirmation that diet may be more effective than conventional therapies requires more evidence.

Regarding the studies mentioned above, a case study of a psoriatic arthritis patient has been reported, in which a whole-food plant-based diet was enough to achieve good disease response, such as decreased scalps, articular pain, stiffness, capsulitis, periods of articular discomfort, and uveitis [24]. In this report, a 40-year-old woman diagnosed in 2003 with psoriatic arthritis, who after 15 years of treatment with methotrexate, changed her dietary habits, adopting a whole-food plant-based diet, was able to discontinue pharmacology treatment based on immunosuppressor drugs due to an absence of disease activity. During the period of the administration of 20 mg methotrexate per week as a base treatment, the patient had a decreased white blood cell count and mouth ulcers as adverse effects. The woman reported that the first improvement in symptoms occurred when avoiding animal-derived foods such as dairy products and red meat; due to the positive change, she adopted a strictly vegan diet with which she reduced the dose of methotrexate to 12.5 mg weekly, compared to the previously implemented gluten-free diet with no clinical improvement. However, total control of the disease was not achieved until a whole-food plant-based diet was fully established. It involved abundant vegetables and fruits, whole grains, no added sugar, oil, or salt, and no refined or processed foods, except soya milk and soya sauce. In addition, in March 2018, after approximately 6 months on a plant-based diet, her blood test results showed an ESR of 10 mm/h and CRP of 2 mg/L [24]. In this regard, evidence suggests that plant-based diets may play a role in modulating inflammation response, and their implementation as a treatment may reduce medication use and avoid anti-inflammatory drug-related adverse effects, but more studies are necessary to substantiate the recommendation for the elimination of medication during psoriasis or psoriatic arthritis.

In another case study, a 47-year-old male with a 28-year diagnosis of severe plaque psoriasis achieved remission of the disease after fast followed by adopting a plant-based diet [25]. His treatment consisted of long-term topical corticosteroids, without achieving full remission of the lesions. The patient stopped using drugs due to concerns about their chronic use and thus adopted a plant-based diet on his own as an alternative therapeutic, which had slight clinical improvement. Thus, the patient decided to undergo a medically supervised water-only fast for 13 days followed by 6 days of refeeding on a vegan and vegetarian diet pattern, until the implementation of a whole-food plant-based diet. The benefits after dietary intervention were a remarkable positive response in the severity of psoriasis plaques, an improvement in nailbed psoriasis pain and arthritis, and significant weight loss with a consequent decrease in the BMI, maintaining it within the normal range. Post-treatment, the patient maintained a whole-plant diet free of salt, sugar, and oil, reporting no new lesions and a continued improvement in existing plaques. Regarding concerns about the safety of this type of diet, the only adverse effect was a mild discomfort in the epigastrium on day 8 of only-water fasting, which was interrupted with vegetable broth and juice; on day 9, he followed fasting to complete the diet intervention [25]. Therefore, this case adds evidence for further research on the therapeutic benefits of plant-based diets and their anti-inflammatory properties in managing skin diseases such as psoriasis.

With the above, it seems that the adoption of a plant-based diet that consists of limited or excluded animal-derived products and increasing the intake of vegetables, fruits, legumes, nuts, and cereal products is beneficial to skin health [7]. As a result, this diet is low in saturated fat, trans fat, and arachidonic acid and high in antioxidants and omega-3 fatty acids, which together with its direct therapeutic effects of reducing inflammation and cutaneous symptoms and decreasing the risk of severe stages of the disease and indirect effects of promoting weight loss result in an improvement in the general life condition of these patients; therefore, using plant-based diets as a potential therapeutic option for the management of some psoriatic patients is recommended (Table 1).

## 3. Association between Plant-Based Diet and Atopic Dermatitis

Atopic dermatitis (AD) is the most common inflammatory skin disease, characterized by erythema papules and pruritic scaly plaques, predominant in skin folds such as hands, neck, and head. Worldwide, it has a prevalence of 2–3% in the adult population [26]. Its etiology is multifactorial, being the result of the interaction of genetic and environmental factors and immunological activity. The pathogenesis of atopic dermatitis describes eosinophils and T-lymphocyte infiltration, apoptosis, changes in the skin microbiota, altered immune responses, and IgE (immunoglobulin-E) sensitization, leading to the deterioration of the skin’s stratum corneum and the epidermal barrier it constitutes [27]. Conventional treatment for AD includes systemic or topical corticosteroids, emollients, anti-inflammatory drugs, antihistamines, and immunosuppressive drugs, such as cyclosporine-A [28]. The first relationship between diet and AD is their association with food allergies; thus, avoiding certain foods seems to be a good therapeutic base [29]. Nowadays, plant-based diets such as the vegetarian diet are recognized as a complementary therapeutic option for AD.

A recent cross-sectional study investigated the relationship between adults with atopic dermatitis and lifestyle factors such as alcohol consumption, stress, obesity, physical activity, sleep duration, and diet. In this study (N = 56,896), it was found that there is no association between diet, specifically vegetarian and vegan diets, and the presence or severity of atopic dermatitis. However, class I obesity, a diet-related disease, was reported as a positively related factor with moderate to severe AD. Data on lifestyle factors were collected between 2006 and 2013. Although past studies have shown the benefits of plant-based diets in the management of AD, the current study did not have the same results. Therefore, more studies seem to be necessary for the complete understanding of the relationship between plant-based diets and AD and therefore to be able to establish this type of diet as part of the treatment of the disease, investigating the effects of a vegetarian diet on atopic dermatitis and the immunological mechanisms of this therapy [30]. In this open-trial study (N = 20), a vegetarian diet significantly decreased the SCORAD (scoring atopic dermatitis, consisting of erythema, edema, crusts, and excoriation) index from 49.9 to 27.4 at the end of the intervention, with a similar effect of ciclosporin-A treatment. The amelioration of skin inflammation went hand in hand with a significant decrease in serological parameters such as LDH5 activity, the number of eosinophils and neutrophils in peripheral blood, and PGE2 synthesis. In the case of eosinophils, which are the main inflammatory cells in AD, their count decreased to half of the basal value, reaching its lowest level 2 weeks after the diet, with a similar effect observed with neutrophils. This cell reduction was prior to the observation of skin improvement, positioning this as the first mechanism in the pathway to suppress inflammation of the skin. In addition, the production of PGE2 by peripheral blood mononuclear cells was monitored, since its overproduction is characteristic in patients with AD, and the inhibition of PGE2 synthesis was observed [31]. Conversely, serum IgE levels did not change during the intervention; however, they decreased after the study period, in which also the number of peripheral eosinophils returned to the normal value. In the study, the intervention was a vegetarian and low-energy diet with an energy intake of 1085 kcal, which includes fresh vegetable juice, brown rice with kelp powder, tofu, and sesame paste, given for 2 months; no vegetarian-diet-related adverse effects were reported [32]. Thus, the observed evidence suggests that a vegetarian diet participates in the mechanism of decreasing AD immunological parameters such as LDH5 activity, the number of peripheral eosinophils, and PGE2 synthesis, which seem to be responsible for skin inflammation, and its suppression results in an improvement in skin symptoms. Nevertheless, more studies and studies with a larger sample size are required to clarify the anti-inflammatory role of plant-based diets in AD.

Moreover, the results showed a significant decrease in the SCORAD index of a total of 19 subjects (N = 19) enrolled, regardless of the degree of severity of the disease, reaching its lowest score at week 2. Other parameters were measured, such as the BMI, systolic blood pressure, LDH-5 activity, and the number of peripheral eosinophils, also finding a significant reduction in all of them. In addition, 8-OHdG, a biomarker of cellular oxidative stress that is excreted in the urine after the DNA repair, was evaluated in severe AD patients, and a marked reduction in its urinary levels was observed, demonstrating that a vegetarian diet is able to reduce the oxidative DNA damage. In this study, the dietary intervention consisted of fresh vegetable juice, brown rice with seaweed powder, tofu, and sesame which represented an energy intake of 55% of the recommended daily allowance but included a high nutritional intake of iron (130% of the daily requirement), vitamin A (150%), vitamin C (250%), and vitamin E (110%) [32]. The main concern with the vegetarian diet and in general all plant-based diets is whether these diets can cause certain degrees of malnutrition; nevertheless, during the study period, no laboratory or clinically serious adverse events were reported. In this regard, vegetarian and low-energy diets may be beneficial in inflammatory skin diseases such as AD, since the results demonstrated their ability to improve the condition of the skin, allowing patients to reduce their SCORAD and therefore decreasing the severity of the disease, in addition to its remarkable role in reducing oxidative damage to DNA by contributing large amounts of nutrients and antioxidants, such as vitamins C and E, present in these type of diets when well planned. Therefore, antioxidants and a low BMI may play a role in reducing skin inflammation [32] Table 1.

## 4. Association between Plant-Based Diet and Acne

Acne vulgaris is the most common form of acne, which is defined as a chronic inflammatory skin disease that affects the pilosebaceous unit. Characteristic skin lesions include comedones, papules, pustules, nodules, and cysts; predominant locations of acne are the face, neck, and chest. Worldwide, it has a prevalence of 9.4%, the most affected population is adolescents with a prevalence of approximately 85%. There are several recognized mechanisms for the development of acne: excess sebum production, hyperproliferation of the bacterium Cutibacterium acnes, hyperkeratinization of the pilosebaceous follicles, and inflammatory mechanisms, in which, insulin-like growth factor 1 (IGF-1) appears to be a key factor in potentiating pathogenesis mechanisms that lead to acne progression and severity by sebum overproduction, as well as sebocyte and keratinocyte proliferation. Furthermore, IGF-1 has a positive feedback loop with androgen levels and is closely related to hyperinsulinemia [33,34,35]. Genetic and environmental factors, such as hormonal factors and diet, are involved in the pathogenesis of acne; for instance, if a certain population adopts a Western diet, it causes the subsequent development of acne. However, the causal relationship between acne and diet is evolving [36,37,38].

In one study, the association between dairy intake and acne was investigated. In this case-control (N = 279 both groups) study, an increased risk of developing moderate to severe acne was reported with the regular consumption of low fat, and whole milk and the intake of eggs had similar results. Unexpectedly, the consumption of chicken was inversely related to acne. However, the specific association between plant-based diets and acne was not investigated in this study, but it was found that the association between the consumption of vegetables, a low BMI, and a follow-up of a diet was higher in the control compared to the case group; however, the type of diet followed was not specified. Consumption habit data were collected for 8 months [39]. The association found between milk intake and acne may be due to its compounds, such as certain amino acids, being able to stimulate the synthesis of IGF-1. Regarding the relationship with the egg, this could be explained by the leucine contained in this food that, through the synthesis of lipids and proteins, increases the activity of sebaceous glands [39]. Therefore, there is a clear association between the consumption of milk and egg and the development of acne, although a relationship with a vegan or vegetarian diet was not found in this study. Following these patterns where foods of animal origin such as milk and egg are avoided could be a useful complementary treatment in acne management.

In another study, the relationship between dietary factors and acne in young adults was determined [40]. The results showed an increased intake of meat, beef, fish, butter, honey, corn, rice, chicken, and pizza in acne patients compared to controls, and conversely, a higher consumption of vegetables and potatoes was described in the control group. Moreover, it was found that among the patients with acne, there was a difference in the amount of vegetables consumed that was greater in mild acne than in severe acne patients. Regarding plant-based diets, significant differences between acne patients and controls were reported. Adherence to a vegetarian diet was observed in more than half of the controls, while in patients with acne, it barely exceeded 10%. In addition, vitamin A supplementation had a statistically significant increase in the vegetarian patients of the control group. This case-control study (N = 460) was carried out for a period of 2 years [40]. Therefore, evidence suggests that the vegetarian diet plays a role in the treatment and prevention of acne since a greater tendency to follow this dietary pattern was observed in the control group in this study. However, more studies are still needed to clarify whether plant-based diets prevent acne, lead to total control of acne, or only contribute to conventional pharmacological treatment.

In this regard, evidence so far about dietary habits and the consumption of certain foods in relation to the development of acne is controversial and contradictory in some cases. For instance, in the studies analyzed in this review, both positive and negative associations regarding chicken and milk intake are reported. However, most studies agree that diets with restrictive patterns, especially foods of animal origin, low-energy diets, and diets with a high consumption of vegetables, provide benefits for acne lesions, improving the condition of the skin of the patients Table 1.

## 5. Plant-Based Diet Effects on Other Skin Conditions

Morgen Smith and colleagues presented a case report from a 63-year-old woman with ulcerations observed on both lower legs and then diagnosed livedoid vasculopathy by biopsy. Livedoid vasculopathy is a rare and chronic skin condition characterized by the development of painful ulcers or sores on the lower extremities, particularly on the ankles and feet. The patient adopted a whole-food plant-based diet (WFPB) as a potential treatment with no other form of treatment. Notably, the symptoms were completely remitted; however, the symptoms recurred due to poor dietary adherence. Finally, the authors speculated that dietary modifications directly impact vascular endothelial health, thereby influencing the likelihood of developing a prothrombotic state [41]. On the other hand, Vini Kamlesh Solanki presented a case report of a 58-year-old female patient who was experiencing symptoms associated with pemphigus vulgaris (PV), a chronic autoimmune blistering disorder. Her symptoms included itching, burning sensations, and the development of fluid-filled blisters throughout her body. Additionally, she had recently been diagnosed with type 2 diabetes mellitus and expressed feelings of malaise, indigestion, and anxiety related to her skin condition. To address her condition, the patient underwent a lifestyle modification program based on yoga and naturopathy. This program spanned a duration of 53 days in 2019, followed by 10 days in 2020 and 15 days in 2021, with subsequent follow-up appointments. The program incorporated different approaches, including hydrotherapy, yoga, a vegetarian diet, herbal preparations, and massage. By the end of 2020, the patient had been successfully weaned off all medications, resulting in complete remission from PV. Considering the complex nature of PV, the comprehensive and patient-focused lifestyle approach described in this case study appears to be advantageous in managing the condition [42].

Another study conducted by Arkom Nongnuch and colleagues proves the beneficial role of plant-based diets in the context of skin. They measured skin autofluorescence (SAF) in 332 adult hemodialysis patients. SAF is an important tool used in hemodialysis to assess the level of advanced glycation end products (AGEs) in the skin. They found that vegetarians had lower skin autofluorescence (SAF) levels compared to non-vegetarians, and they found a negative correlation between SAF and a vegetarian diet observed in both univariate analysis and multiple linear regression analysis. This suggests that a vegetarian diet may lead to a reduction in tissue advanced glycation end product (AGE) deposition, as indicated by SAF levels. It is speculated that by modifying the diet, it may be possible to lower tissue AGE levels and SAF, subsequently reducing the risk of cardiovascular disease (CVD) [43].

In contrast, another study led by Marta Fusano and collaborators compared surgical scars between patients adhering to an omnivore diet and those following a vegan diet through a prospective observational study, enrolling 21 omnivore patients and 21 vegan patients who had undergone surgical excision for nonmelanoma skin cancer. The researchers assessed postsurgical complications and scar quality using the modified Scar Cosmesis Assessment and Rating (SCAR) scale. They found that wound diastasis was more prevalent among vegans, and after a 6-month period, vegan patients had higher modified SCAR scores compared to omnivores, indicating more pronounced scar spread, a higher incidence of atrophic scars, and a poorer overall impression. Finally, they concluded that a vegan diet may have a detrimental impact on the outcome of surgical scars [44] Table 1.

## 6. The Role of Plant-Based Functional Foods on Skin Health 

**Figure 2:**
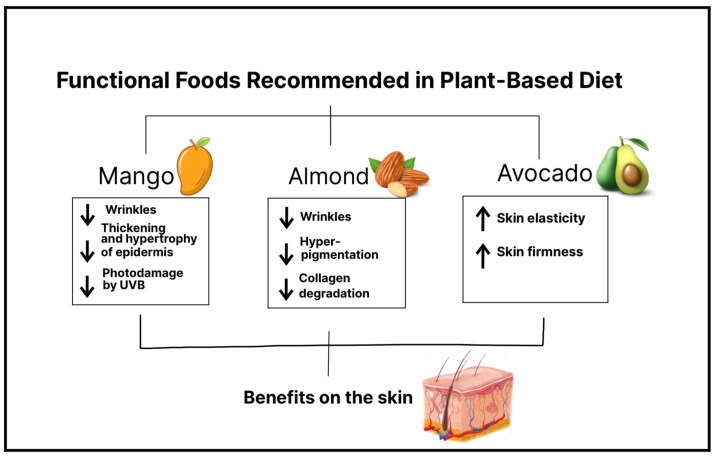
Specific plant-based functional foods recommended to add to a plant-based diet for greatest benefits. The image highlights three functional foods—mango, almond, and avocado—and their positive impact on skin health. Mango stands out for its ability to reduce wrinkles and the thinning and hypertrophy of the epidermis and protect against UVB damage. Almonds are known to reduce wrinkles, hyperpigmentation, and collagen degradation. Avocado, on the other hand, promotes increased skin elasticity and firmness. In summary, the image emphasizes how these three plant-based functional foods can be excellent additions to the diet, enhancing skin health, reducing signs of skin aging, and improving overall appearance.

The face is the part of the body that is most exposed to sunlight, and finding ways to protect and repair it is becoming a topic of increasing interest. Photoaging is induced by continuous exposure to UV radiation, causing collagen degradation and a lack of elastin fibers in the dermis and, as a result, the formation of wrinkles, a decrease in elasticity, and dyspigmentation of the skin [45]. In general, people are increasingly interested in skin care and rejuvenation, and certain populations are especially looking for diet-based therapeutic options. Nowadays, it is known that diet can modify skin health. In accordance with the International Life Sciences Institute, “functional foods” are foods that provide health benefits beyond basic nutrition, due to the effectiveness of their active substances [11]. Therefore, in a plant-based diet, we can find a wide variety of plant-based functional foods, and if they are consumed regularly and in a proper amount, it is possible to observe their beneficial effects on the skin [46] (Figure 2).

### 6.1. Mango

Mangos are a rich source of vitamin C, B-carotene, xanthones such as mangiferin, and phenolic acids, the main ones being gallic acid, chlorogenic acid, protocatechuic acid, and vanillic acid [47]. Moreover, vitamin C is a known antioxidant, and it is found in high concentrations in skin cells in the human body. Among the properties of vitamin C or ascorbic acid is its ability to stimulate collagen and elastin synthesis, protect against oxidative stress, and reduce damage caused by UV-induced reactive oxygen species (ROS), thus preventing photoaging. B-carotene is a precursor of vitamin A, both of which can provide photoprotection and prevent photodamage due to increased epidermal proliferation [48]. In addition, certain types of mangos, specifically Ataulfo mango, contain more than the daily diet requirement of vitamin C [49].

Regarding the protective properties of mango on the skin, a study reported that mango extract obtained from dried mango reduces the formation of wrinkles induced by UVB radiation, improves wrinkles, inhibits the loss of collagen fibers, and decreases the thickening of the epidermis, as well as its hypertrophy. Therefore, the results confirm a clear photoprotective and antiaging activity of mango, in which mangiferin seems to play a key role. This study was carried out in mice, and the mango intervention was performed by oral administration. This evidence leaves a foundation for future mango intake interventions in humans [45].

In another study, the effects of Ataulfo mango intake on the development of facial photodamage were investigated. This clinical-trial study (N = 28) reported evidence that 0.5 cups of frozen Ataulfo mango consumption can decrease facial wrinkle depth and severity, the results were observed in an 8-week to 16-week period, and serum triglycerides decreased compared to basal values. This study also demonstrated that higher amounts of mango (1.5 cups) significantly increased facial wrinkles. The beneficial effects reported may be due to mango compounds such as mangiferin, carotenoids, and flavonoids, while the unexpected effects of high amounts of mango may be due to the total sugar intake resulting in the glycation process of collagen fibers and therefore the disruption of its structure. The concerns in this intervention were mainly the increase in blood glucose levels; however, there were no changes in this parameter [49]. Thus, the results showed that mango decreases wrinkles and photodamage induced by UVB due to the effects of a whole active compound complex, emphasizing the importance of consuming adequate amounts of foods and avoiding their overconsumption which can worsen skin conditions. Of note, evidence on the favorable effects of mango intake on the skin is still limited; however, the already known antioxidant properties of its compounds, together with recent promising developments, are sufficient to promote its intake in a balanced and adequate diet.

Furthermore, in another study, it has been shown that the antioxidant properties and bioavailability of mango phenolic acids are preserved whether the pulp is ingested or processed into juice, with the juice presentation being the one in which their properties may be enhanced [50]. This evidence suggests that people can consume mango in various presentations without concerns about diminishing its properties.

### 6.2. Almond

Almonds are a rich source of alpha tocopherol (or vitamin E), fatty acids, and polyphenols and are thus a food with antioxidant properties [51]. Negar et al. investigated the effects of almond intake on the severity, depth, and length of facial wrinkles, and the results showed a significant improvement in wrinkles. In this control-trial study (n = 31), women consumed 2.1 oz (340 kcal) of almonds packaged for 16 weeks. During the intervention period, it was reported that there was no increase in sebum production in the almond group or any other adverse effect related to its intake [52].

Another study proved that daily consumption of almonds significantly decreased average wrinkle severity and the intensity of facial pigmentation without an increase in sebum production in the face in 56 postmenopausal women [53]. The beneficial effects of alpha tocopherol against skin aging could be due to vitamin E consumed through almond intake which has antioxidant and photoprotective properties and thus a protective effect against free radicals and the damage they cause since vitamin E is incorporated into the cell membrane. Vitamin E can also potentially prevent collagen degradation, thanks to a decrease in the activity of matrix metalloproteinases. Therefore, an adequate daily intake of almonds can be a potential alternative to prevent or decrease the progression of wrinkles and facial pigmentation and thus skin aging.

### 6.3. Avocado

Recent studies have provided evidence that avocados have beneficial properties due to the food’s compounds: carotenoids, monounsaturated fatty acids, and phenols. The well-known beneficial properties of avocado on the skin, such as increased skin elasticity, include its topical application as a cream, but it has recently been demonstrated that oral intake of avocado also has skincare effects [54]. For instance, Henning et al., in a pilot study (N = 39), reported significant changes in facial skin in overweight women who added the consumption of an avocado to their daily diet. The results were an improvement in skin elasticity and skin firmness in the forehead and under-eye location. There were no adverse effects related to avocado intake such as weight gain or increased production of facial sebum. This improved skin status might be a result of a mechanism in which the expression of certain genes, such as collagen and elastin genes, is induced after making dietary modifications [55]. Thus, the evidence found suggests that the addition of avocados to the daily diet has a potential positive impact on improving and maintaining the elasticity and firmness of the skin, or delaying aging.

## 7. Association between Plant-Based Diet Alternatives and Gut Microbiome

The effects of different types of diets on the skin have gained great interest in the last decades. However, it is difficult to evaluate the impact that diets have directly on the skin because they also have significant effects on the gut microbiome, which can also influence skin health [56]. Indeed, extensive evidence has demonstrated a bidirectional interaction between the intestine and the skin either to maintain their homeostasis or to alter their biology [57]. For instance, animal models of patients with common skin disorders such as psoriasis and atopic dermatitis, as well as other less common conditions such as rosacea, show distinct gut microbial profiles [58].

The gut microbiome is the largest endocrine organ, located between food intake and various body organs such as the skin. It produces several metabolites including short-chained fatty acids (SCFA), neurotransmitters, tryptophan derivatives, and bile acids, among others. These metabolites influence gut physiology, including gut barrier integrity, and can also reach distant organs generating pleiotropic effects [59]. Interestingly, skin health has been associated with the integrity of the intestinal barrier [60]. Moreover, some bacterial metabolites show immuno-modulating potential on distant organ sites such as the skin which is currently a growing field of research. While some dietary metabolites can be absorbed directly into the skin [61,62], others do so via gut microbial metabolism, both of which can potentially contribute to skin health and various skin disorders and diseases [63,64].

Among numerous other factors, diet strongly influences the composition of the gut microbiome and, in this way, the organism’s metabolic and immunological functions [56]. Changes in macronutrients, as well as their source (plant or animal), may particularly affect the diversity of the gut microbiome [65]. In addition, the amount and source of dietary fiber can have a major impact on the composition and diversity of the human gut microbiota [66]. To illustrate, the consumption of whole grains or cereal fibers can induce a higher abundance of *Bifidobacterium*, which is decreased in distinct pathologies, including atopic dermatitis [67,68].

The importance of the host dietary patterns on the gut microbiota has also been demonstrated in studies that observe a greater bacterial diversity, as well as a greater abundance of potentially beneficial bacteria, with a high-quality diet or a diet rich in “healthy foods” [69,70,71]. Other studies show that Mediterranean or vegetarian/vegan diets, enriched in plants (an important source of natural fiber) and omega-3 fatty acids and low in saturated fat and animal protein induces an increase in dietary-fiber-metabolizing bacteria that produce SCFA [72]. In contrast, the consumption of a high-fat diet induces alterations in the composition of the gut microbiota, termed dysbiosis, that by increasing the release of pro-inflammatory cytokines promotes higher intestinal permeability [73].

Decades ago, studies reported an association between the consumption of Western diets and a variety of skin diseases such as acne, atopic dermatitis, and psoriasis [74,75,76]. Thus, it has been described that the infiltration of molecules, derived from an increased intestinal permeability, further introduces proinflammatory mediators into the circulation which can lead to the disruption of skin homeostasis and thus contribute to the physiopathology of these diseases [73]. Moreover, bacterial metabolism derived from the consumption of Western-type diets can result in the production of harmful metabolites, such as p-cresol, that are also associated with impaired epidermal barrier function [58].

Remarkably, in the consumption of plant-based diets, microbial metabolism release and transform phytochemicals with anti-inflammatory and antioxidant functions [77]. Moreover, the production of SCFA associated with the consumption of the Mediterranean diet (also rich in plants) can have favorable effects on gut barrier integrity [78]. SCFA, particularly butyrate, are also able to improve the skin barrier and relieve skin inflammation [79]. In fact, low levels of fecal SCFA have been observed in patients with eczema and atopic dermatitis, and interventions with SCFA-producing probiotics are able to ameliorate inflammation in other skin conditions [80].

Overall, the latter evidence underscores the importance of the diet–gut–skin axis. In fact, it has been proposed that skin inflammation is also a consequence of changes in the intestinal microbiome caused by specific diets [81]. For example, in dermatitis herpetiformis, a cutaneous manifestation of celiac disease, patients can palliate their skin rashes when consuming a gluten-free diet [82].

**Figure 3:**
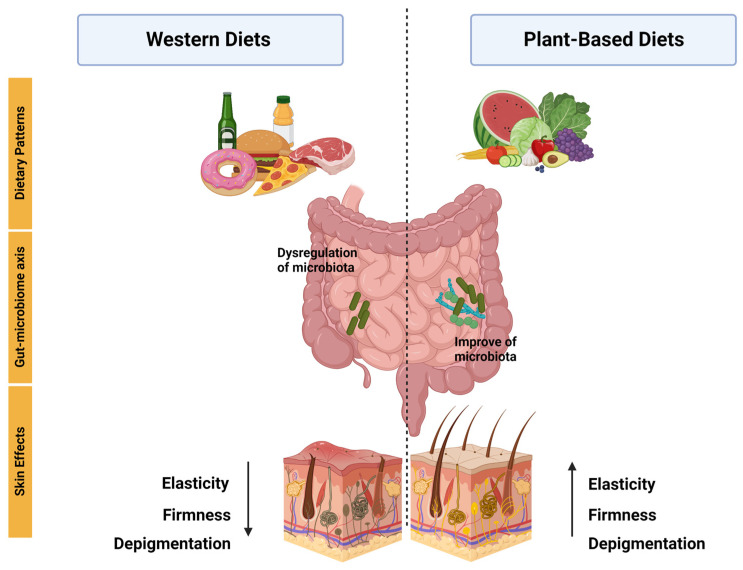
Following a plant-based diet and maintaining it over time have demonstrated positive effects on the skin. The gut-microbiome axis has been demonstrated to be dysregulated in Western diets. In contrast, plant-based diets have been shown to enhance the composition of the microbiome, leading to positive effects. Western diets, however, have been linked to a reduction in skin elasticity, firmness, and depigmentation. Conversely, plant-based diets have demonstrated an opposite effect, as indicated by the arrow, by promoting healthier skin.

Research has suggested that plant-based functional foods may have a beneficial impact on inflammatory diseases, specifically by improving skin health. This improvement has been linked to the interaction between the intestinal microbiota and a diverse range of bacteria that are specific to these skin diseases, including psoriasis, atopic dermatitis, and acne (Figure 3).

For example, in a 12-week crossover design study, 27 participants consumed 100 kcal/day of either mangos or low-fat cookies with a washout period of 4 weeks. Interestingly, mango consumption increased the abundance of *Prevotella maculosa*, *Corynebacterium pyruviciproducens*, and *Mogibacterium timidum* while decreasing the abundance of *Prevotella copri* [83]. *Prevotella copri* has been shown to be increased in subjects with psoriasis; thus, it will be interesting to evaluate whether mango consumption could have a beneficial effect on psoriasis and other skin diseases [84].

In a study where the effect of almond intake was observed in 48 subjects with a daily dose of toasted almonds (56 g) or fructooligosaccharides (as a control) for 6 weeks, it was observed that there was an increase in *Bifidobacterium* spp. populations as a consequence of almond or almond skin supplementation. *Bifidobacterium* abundance has been shown to be decreased in patients with atopic dermatitis; thus, almond intake could have a potential beneficial effect on atopic dermatitis [68,85].

Finally, it is important to consider that skin microbiota is significantly regulated by components from the gut microbiome [76], involved in the physiopathology of several dermatoses [74], and that the maintenance, prevention, or restoration of skin health can be achieved with the consumption of prebiotics and probiotics due to their beneficial effects in the gut microbiome [75], reducing intestinal inflammation caused by alterations in the intestinal microflora by diets. Taking into account all of the above, treating the microbiome with supplementations, as well as with proper diets, may contribute to the restoration of the intestine microflora, helping to improve certain skin diseases.

**Table 1:** Summary table of the main research in which plant-based diets are associated with inflammatory skin diseases.

Study Design	Participant Characteristics	Intervention Details	Outcomes	Ref
Exploratory survey study	1206 psoriasis patients. The mean age of the sample population was 50.4, and 73% of respondents were female.	The survey contained 61 questions; the first 30 questions were from National Health and Nutrition Examination Survey; the last 31 questions focused on patient-report skin responses to dietary changes.	72% of Pagano, 70% of vegan, and 68.9% of Paleolithic diets reported favorable skin response. The addition of fruits to the diet was protective against psoriasis, OR 0.22, *p* < 0.0001.	[21]
Assessor-blind, randomized controlled trial	303 patients between 18 and 80 years, overweight or obese, with moderate to severe chronic plaque psoriasis.	Each participant underwent an introductory session with a dietitian. Subjects were instructed to perform sessions of continuous aerobic physical exercise for at least 40 min three times a week. Compared how psoriasis severity was affected by either a 20-week quantitative or qualitative dietary plan associated with physical exercise for weight loss.	Reduction of PASI score by 48% in the dietary intervention and 25.5% in the information-only.	[23]
Cross-sectional study	56,896 participants. The mean age was 55.8, and 22,577 were female.	First, the researchers sent out a digital questionnaire focused on atopic dermatitis (AD). After collecting the responses, they administered another questionnaire to extract data on lifestyle factors, such as smoking, alcohol consumption, stress, obesity, physical activity, and diet (participants were dichotomized into vegetarian/vegan and nonvegetarian/nonvegan), and finally analyzed the association between lifestyle factors and presence of AD.	No associations were observed with abdominal obesity, physical activity, diet quality, or a vegetarian/vegan diet.	[30]
Controlled clinical trial	19 patients with atopic dermatitis. The mean age was 25.5.	The patients were exposed to a low-energy diet, no pharmacotherapy. The SCORAD indices were noted by 2 dermatologists. Blood and urine samples were collected at weeks 0.4 and 8. The 24 h void urine was collected and kept frozen at −80 °C.	SCORAD significant reduction from 6.1 ± 2.8 at week 0 to 2.7 ± 2.8 at week 8.	[32]
Clinical trials	Twenty patients (6 males and 14 females) aged 15 to 36 years (average: 25 years) with AD varying in severity from mild to serious. All patients fulfilled the criteria for atopic dermatitis (AD) established by Hanifin and Rajka.	All patients followed the same meal plan throughout the study. The diet included a glass of fresh vegetable juice for breakfast, followed by brown rice porridge, kelp powder, tofu, and sesame paste for both lunch and dinner. In addition, a daily intake of 2.5 g of non-refined salt was included in the diet. Instead of regular water, the patients were provided with persimmon leaf tea. This treatment regimen was followed for two months.	Reduction in SCORAD index from 49.9 ± 18.6 to 27.4 ± 16.8 (*p* = 0.001), diminution in peripheral eosinophil count 423 ± 367 to 213 ± 267 (*p* = 0.01), reduction in monocyte PGE2 synthesis 2886 ± 1443 to 1390 ± 773 (*p* = 0.001).	[31]
Case-control cross-sectional questionnaire study	57 acne vulgaris patients as cases and 57 participants as controls. Aged 14 and above and were seeking medical consultations at a private clinic.	The Comprehensive Acne Severity Scale (CASS) grading system was used to grade acne severity. Cases were defined as patients with CASS grade of two to five and controls with CASS grade 0 or 1. Thereafter, controls were recruited and matched by age, gender, and ethnicity. A self-administered questionnaire was used to collect information regarding the participants’ dietary intake and cigarette smoking habits.	No significant associations were found between dietary patterns and acne; on the other hand, they only found an association between milk and chocolate consumption and acne.	[36]
Case-control study	279 acne patients and 279 controls aged 10–24 years.	Acne severity was determined by a dermatologist using the Global Acne Severity Scale. Epidemiological data were collected with a pre-structured questionnaire. The food consumption habits were recorded using a food frequency questionnaire. Investigated food included whole milk, low-fat milk, cream of milk, ice cream, cheese, chocolate, cake, potatoes, fresh fruit, fresh vegetable, meat, chicken, and egg. Consumption habit data were collected for 8 months.	The study found that consuming whole milk at least three days a week was significantly associated with moderate to severe acne (OR = 2.36, 95% CI, 1.39–4.01). The association was slightly weaker for low-fat milk (OR 1.95, 95% CI, 1.10–3.45).	[39]
A prospective case-control study	460 subjects of both sexes, 230 patients with acne vulgaris, and 230 healthy volunteers were included as controls.	Acne severity in patients was classified as mild, moderate, and severe according to the classification of the American Academy of Dermatology. Two formulated tools were used in the study. First tool: interview questionnaires that included two parts (sociodemographic data, anthropometric measurements, and questions related to food habits, drug intake, and diet history). Second tool: a recommended diet regimen.	Significantly decreased consumption of vegetables was noticed among severe and moderate acne patients compared with mild ones.	[40]
Clinical-trial study	Healthy postmenopausal women aged 50 to 70 (N = 28). Inclusion criteria were Fitzpatrick skin type I, II, or III and a body mass index (BMI) between 18.5 and 35 kg/m^2^.	Participants were randomized by block design into an open-label, two-arm parallel clinical trial consuming either 85 g or 250 g of Ataulfo mango, four times per week for 16 weeks.	0.5 cup frozen Ataulfo mango consumption decreases facial wrinkle depth and severity and photodamage induced by UVB. Serum triglycerides decreased. A higher amount of mango (1.5 cups) showed the contrary effect.	[49]
Randomized clinical trial	Thirty-one participants (all postmenopausal females) with a Fitzpatrick skin type 1 or 2.	Consisted of a total of five study visits following a 4-week dietary washout period: baseline, 4, 8, and 16 weeks. The patients were randomized into two intervention groups: almond group and control group. Almond group received an average of 340 kcal/day of almonds.	There were no significant differences in sebum production and transepidermal water loss between the almond and control groups. Wrinkle severity and wrinkle width were significantly decreased in the almond group compared with the control intervention.	[52]
Randomized- blinded	56 postmenopausal women with a Fitzpatrick skin type I or II.	A total of 56 female participants were randomly assigned to two intervention groups: the almond group, which received almonds, and the control group, which received a calorie-matched snack. In the almond group, the dose of almonds provided accounted for 20% of the total energy in the participants’ diet. The almond snack group received approximately 2 g of sugars per day, whereas the control snack group received approximately 8 g of sugars per day that was additional to their regular food intake. 24 weeks.	Reduction in the average severity of wrinkles compared to the baseline. Additionally, intensity of facial pigmentation decreased in the almond group.	[53]
Randomized parallel group	39 women with a Fitzpatrick skin type II–IV.	The patients were randomized into two intervention groups: avocado and control. The participants either consumed one avocado daily or no avocado based on randomization performed. 8 weeks.	Daily consumption of one avocado per day for 8 weeks improved firmness and elasticity and reduced the tiring of repeat stretching of the forehead skin.	[55]
Case-control	30 patients with AD (according to the criteria for the diagnosis of AD established by the Japanese Dermatological Association) less than 20 years of age and age- and sex-matched control subjects (68 healthy individuals).	A questionnaire survey was conducted for 1 week, and then fecal specimens and 24 h skin secretion specimens were collected from all subjects. Fecal microflora, fecal IgA levels, and IgA concentrations in the skin were analyzed. The data for the 2 groups (i.e., patients with AD and healthy control subjects) were compared.	The counts of *Bifidobacterium* were significantly lower in patients with AD than in healthy control subjects (9.75 ± 0.68 vs. 10.10 ± 0.50, *p* < 0.05). Percentages of *Bifidobacterium* were significantly lower in patients with severe skin symptoms than in those with mild skin symptoms (40 ± 6% vs. 19 ± 6%, *p* < 0.05). In addition, the frequency of occurrence of *Staphylococcus* was significantly higher in patients with AD than in healthy control subjects (83% vs. 59%, *p* < 0.05).	[85]
Case-control	21 psoriasis patients from a Brazilian referral dermatology service and 24 controls.	A stool sample was collected from each participant at the time of inclusion in the study, and the samples were analyzed by sequencing the 16S rRNA gene.	Patients with psoriasis showed higher levels of the *Dialister* genus and the species *Prevotella copri* compared to the control group. Conversely, there was a decrease in the *Ruminococcus, Lachnospira*, and *Blautia genera*, as well as the *Akkermansia muciniphila* species, in the psoriasis group compared to the control group. Additionally, individuals with psoriasis had lower diversity in their gut microbiota compared to the control group.	[84]
Case report	63-year-old woman with ulcerations on both lower legs and diagnosed livedoid vasculopathy by biopsy.	The patient adopted a whole-food plant-based diet (WFPB) as a potential treatment with no other form of treatment.	The symptoms were initially completely resolved, but they reappeared later due to a lack of adherence to a proper diet.	[41]
Case report	58-year-old female patient with pemphigus vulgaris and type 2 diabetes mellitus.	Incorporated a change in lifestyle with different approaches, including hydrotherapy, yoga, a vegetarian diet, herbal preparations, and massage.	The patient was successfully weaned off all medications and had complete remission from PV.	[42]
Cross-sectional	They measured skin autofluorescence (SAF) in 332 adult hemodialysis patients who were dialyzing in north central London.	SAF was measured in 332 adult dialysis patients.	SAF was lower in the twenty-seven vegetarians than it was in the non-vegetarians.	[43]
Observational retrospective	21 omnivore patients, 21 vegan patients, with surgical excision of melanoma.	The assessment of postsurgical complications and the quality of scars was conducted using a modified version of the Scar Cosmesis Assessment and Rating (SCAR) scale.	Wound diastasis was more frequent in vegans (*p* = 0.008). Higher modified SCAR score than omnivores (*p* < 0.001), worst scar spread (*p* < 0.001), more frequent atrophic scars (*p* < 0.001), and worse overall impression (*p* < 0.001) were observed in vegans.	[29]

## 8. Conclusions

This review article summarizes the benefits and concerns of a plant-based diet for the treatment and maintenance of skin health. Evidence suggests that plant-based diets can be appropriate for individuals during all stages of the life cycle and have several benefits for skin health and certain skin diseases after implementation and maintenance over time. Many studies have been able to remove the misconception about plant-based diets, such as the perception that following them leads to a state of malnutrition. Nowadays, it is known that nutritional intake is a factor in the aging process, as well as for healthy skin. The positive effects that have been reported range from preventing photoaging, improving skin firmness and elasticity, and decreasing skin pigmentation and facial wrinkles to being a fundamental pillar to total control in inflammatory skin diseases such as psoriasis, atopic dermatitis, and acne. In particular, some functional plant foods when incorporated into the diet have beneficial effects on skin health. Furthermore, both plant-based diets, as well as some fruits and vegetables, impact the microbiome. In this regard, plant-based diets have antioxidant and anti-inflammatory properties, due to the foods and their active compounds. Therefore, certain plant foods can be considered as a non-pharmacological treatment option with fewer adverse effects for specific skin conditions and skincare. However, more studies that focus on exploring the potential beneficial effects of plant-based diets and plant-based functional foods are needed.

## Data Availability

Not applicable.
